# Simulation study of a practical approach to enhance cadmium removal via biological treatment by controlling the concentration of MLSS

**DOI:** 10.1038/s41598-023-50843-5

**Published:** 2024-01-19

**Authors:** Basim K. Nile, Ahmed M. Faris, Hasan F. Alesary, Nadhir N. A. Jafar, Hani K. Ismail, Muhammad Abdulredha, Maad F. Al Juboury, Waqed H. Hassan, Luma M. Ahmed, Hussein Rasool Abid, Stephen Barton

**Affiliations:** 1 Engineering College, University of Kerbala, Karbala, 56001 Iraq; 2 Kerbala Sewerage Directorate, Kerbala, 56001 Iraq; 3 Department of Chemistry, College of Science, University of Kerbala, Karbala, 56001 Iraq; 4 Al-Zharaa University for Women/Al-Zharaa Center for Medical and Pharmaceutical Research Sciences, Karbala, Iraq; 5 Department of Chemistry, Faculty of Science and Health, Koya University, Koya, KOY45 Kurdistan Region-F.R. Iraq; 6 Department of Civil Engineering, College of Engineering, University of Kerbala, Kerbala, 56001 Iraq; 7 University of Warith Al-Anbiyaa, Kerbala, 56001 Iraq; 8 Environment Health, Applied Medical Sciences College, University of Kerbala, Karbala, Iraq; 9 School of Life Sciences, Pharmacy and Chemistry, Kingston University London, Kingston-Upon-Thames, Surrey, UK

**Keywords:** Environmental sciences, Chemistry, Engineering

## Abstract

The fate of cadmium at the Muharram Aisha wastewater treatment plant in Karbala governorate, Iraq was studied using the TOXCHEM model. Cadmium, a known carcinogen, and is considered one of the most dangerous heavy metals and high concentrations, greater than permissible limits, were found in the treated wastewater. The plant operates using an activated sludge system and this was modeled via TOXCHEM with a sensitivity analysis carried out on the extended aeration system. Prior to analysis, the model was calibrated and validated for cadmium, with the adjustments leading to a mean square error (RMSE) and correlation coefficient (R) of 0.0001 and 0.81, respectively. The mass balance of cadmium in the Muharram Aisha treatment plant was found to be 4832.44 g/day (37.1726%) in the treated wastewater and 8164.52 g/day (62.804%) in the sludge, which indicated that the mix liquor suspended solid (MLSS) was the most sensitive factor. The sensitivity to cadmium was analyzed via MLSS in the extended aeration system and the results o indicated that the higher the MLSS concentration (mg/L), the greater the removal of cadmium in the treated wastewater. It was found that increasing the MLSS through a biological treatment method reduced the concentration of cadmium without the need for additional of any (potentially harmful) chemical treatments. The plant was subsequently operated for a period of 5 months with the MLSS increased from 1500 to 4500 mg/L, and this reduced the concentration of cadmium in the wastewater from 0.36 to 0.01 mg/L as a consequence. This research demonstrates how the novel application of TOXCHEM can be a useful tool in the reduction of heavy metal contamination in the environment.

## Introduction

Industrial effluent from electroplating, welding, batteries industries, paints and plastics, fertilizers, and other metal-processing industries are leading to an accumulation of toxic metals in surface and subsurface water. These metal ions can then be absorbed by microorganisms, forming complex compounds that are non-biodegradable in vivo; and can be introduced into the food chain through routes such as agricultural irrigation and contamination of drinking water. Human exposure to acute or chronic cadmium (Cd) levels can lead to various conditions such as kidney damage, high blood pressure, osteoporosis, testicular tissue destruction, and the disruption of red blood cell production^1^. Cadmium has been classified as a carcinogen by the World Health Organization and given a maximum limit for safe drinking water of 0.005 mg/L^1,2^, while Iraqi legislation specifies that the concentration of cadmium in the treated wastewater should be less than 0.01 mg/L. A range of simple, sensitive, selective, and accurate analytical methods have been developed to determine the presence and concentrations of such species. These include absorption/emission spectroscopy, fluorescence spectrometry, electrochemical technologies and inductively coupled plasma–mass spectrometry^3–5^.

In terms of remediation, technologies such as ion exchange, adsorption, and membrane separation have recently been developed for use in water pollution control^4,6^. However, these methods have inherent limitations such as low efficiency, high energy usage and operational difficulties. Furthermore, the high cost and the possibility of sedimentation of other wastes, can limit their industrial applications^7^.

Biological methods are considered a particularly attractive alternative to physical–chemical processes for removal of a broad range of heavy metal ions due to their low cost, rapid response and eco-friendliness^8^. Recently, several technologies such as ion exchange, adsorption, and membrane separation used in water pollution control^5,9^. However, these methods have inherent limitations such as difficult operation, low efficiency, and high energy.

The biological method is based on activated sludge and depends on the concentration of MLSS inside the biological reactors. The biomass is the largest and most important and largest part of MLSS^10^ and contains slime, which forms a flocculant. Heavy metal ions are absorbed by the biomass and transported to the flocculant. The greater the biomass within the biological reactors, the greater the absorption processes^11^. Operations to control the fate of heavy elements in a practical way and conduct laboratory tests are time-consuming and expensive^12^. Therefore, the fate of the cadmium was modelled using one of the more reliable simulation programs. There are several simulation programs in the field of wastewater treatment plants, namely GPS-X, SWIMU works, Mantis AI. TOXCHEM, and KABDET works^13^. After reviewing these programs, it was noted that the one that best supports the field of heavy metals in wastewater is the TOXCHEM Model. TOXCHEM, a replacement for the Water8 (Water9) software created by the EPA, was developed in the early 1990s to address some of the former software’s drawbacks, involving enhanced mass transfer techniques, sorption of pollutants to MLSS, and a compound database with critically reviewed chemical, physical, and biological properties^14^. The removal mechanisms of stripping and volatilization, biodegradation, and sorption are all part of the foundational mass transfer equations and mass balances that form the basis of the TOXCHEM Model. The fate of any synthetic chemical substance for which the physical, chemical, and biodegradation properties are known can thus be ascertained using this method^15^. Previous studies using the TOXCHEM model include the fate and emission of hydrogen sulfide gas from a sewage treatment plant using the extended aeration process and the fate and emission of phenol in a MBBR treatment plant^15,16^. A study of the fate and emission of various volatile organic compounds in the treatment plant in Tabriz, Iran, was carried out using a TOXCHEM model^17^.

A major challenge in this study was the high concentrations of cadmium in the treated wastewater, due to factories that allow the release of this element into the environment. In order to comply with strict local laws, the aim was to ensure the reduction of these concentrations as quickly as possible without the need for any chemical additives. Sensitivity analysis was used in the TOXCHEM model to determine the concentration of MLSS that optimally reduces cadmium concentrations, and then the determination of how to operate the plant practically based on the simulation data.

## Materials and methods

### Site location and description

**Figure 1:**
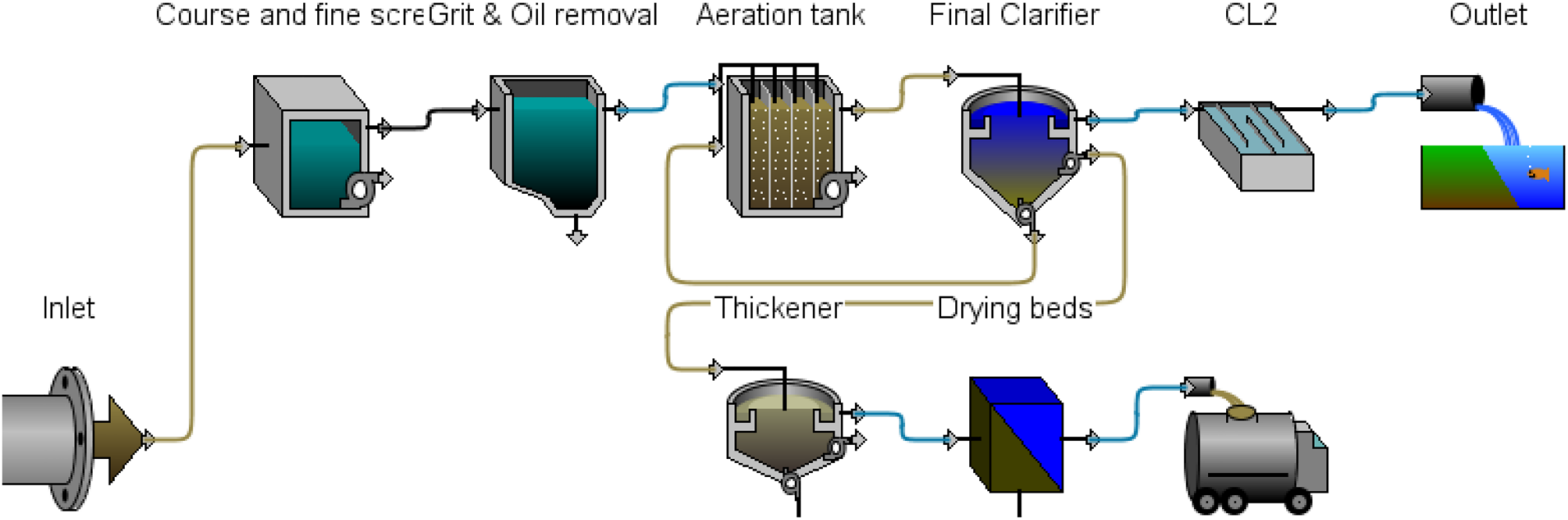
Schematic diagram of Muharram Aisha WWTP.

Muharram Aisha WWTP is located in Karbala Governorate, about 110 km south of the Iraqi capital, Baghdad. The geographical coordinates of the station’s location are 32° 31′ 41.4516′′ N and 44° 13′ 12.2664′′ E. The plant serves some 50,000 people with a capacity of 12,000 m^3^/day. The plant operates an extended aeration activated sludge system, as shown in Fig. 1. The plant operates via several stages, as follows:

- *Preliminary treatment stage*, which includes the lifting station, coarse and fine screens, grinder, and a grit and oil removal basin. It does not contain primary treatment (primary sedimentation tanks) due to the processes that depend on extended aeration.
- *Secondary treatment stage* includes aeration ponds and secondary sedimentation tanks.
- *Tertiary treatment stage*, based on chemical disinfection by chlorination.
- *Sludge treatment stage* includes thickening basins and drying beds.

The plant operates according to the data shown in Table 1.

**Table 1:** Operational conditions of Muharram Aisha WWTP.

Parameter	Value
Flowrate (Q)	13,000 m^3^/day
Sludge retention tank (SRT)	25 days
Hydraulic retention time (HRT)	13 h
Mix liquor suspended solid (MLSS)	1500 mg/L
Food/microorganism (F/M) ratio	0.27 kg BOD/kg MLVSS day
Dissolved oxygen (DO)	2–3 mg/L
Return activated sludge (RAS)	50%
Sludge volume index (SVI)	80 mL/g
MLVSS/MLSS	0.79

### Sampling and analysis

Samples were collected from the management of the Muharram Aisha WWTP during different seasons in 2022. Prior to subsequent evaluation, all parameters were tested according to the standard methods for the examination of water and wastewater^18^, as shown in Table 2. In this study, the element cadmium was evaluated according to the prevailing Iraqi regulations used for irrigation which allows treated water to flow directly back into rivers.

**Table 2:** Chemical analysis and characteristics of Muharram Aisha WWTP.

Parameter	Inlet Concentration	Outlet concentration	Standard Iraq^14^ and River Conservation Legislation No. 25 of 1967
pH	7.2	7.4	7
COD (mg/L)	500	35	100
BOD_5_ (mg/L)	330	10	40
TSS (mg/L)	250	8	60
NO_3_ (mg/L)	0	45	50
NH_4_^+^ (mg/L)	22	0.5	1
PO_4_-P (mg/L)	5	2	3
H_2_S (mg/L)	35	ND	3
SO_4_ (mg/L)	400	450	600
Oil and grease (mg/L)	40	3	4
Cadmium (mg/L)	1	0.36	0.01

### TOXCHEM model setup

In this study, TOXCHEM 4.1 was used to simulate the fate and emission of cadmium in a treatment plant that operates via a bacterial suspended growth technique^17^. The TOXCHEM model is one of the most popular models used to track the existence and emission of volatile organic compounds (VOCs) and heavy metals in wastewater. Cadmium can be found out via two paths in wastewater treatment plants. In the first, it is partially adsorbed by the biomass or the so-called MLSS concentration, whilst in the second path it might be emitted into the effluent of wastewater.

### Cadmium adsorption on MLSS

The mixed liquor suspended solids (MLSS) concentration is typically stated in milligrams or grams per liter. In the activated sludge process, mixed liquor is a mixture of raw or settled wastewater and activated sludge that is kept in an aeration tank. The suspended growth process in a wastewater treatment plant is managed by MLSS^19^. MLSS is characterized as a good property for heavy elements adsorbed through the slime present in the floc bacteria that abound in the activated sludge system. Cadmium is adsorbed from the liquid phase to the solid phase and the adsorption process in the wastewater can be described by the following equation^14^:

1 $${C}_{x}={k}_{p}C,$$

where C_x_ = concentration of contaminant in the solid phase, mg/kg (µg/g), K_p_ = sorption partition coefficient, m^3^/kg (L/g), C = concentration of contaminant in the liquid phase, mg/m^3^ (µg/L).

### TOXCHEM model calibration and validation

Before any model can be adopted, calibration is required^20^. After entering all the necessary data into the model as shown in Table 4, a calibration was carried out for the average for spring and summer, and a verification procedure for the average for the autumn and winter. After changing the default values within the model, results were obtained that were very close to real-world measurements. The predicted values were compatible with the real results, but it is statistically verified via determination of their root mean square error (RMSE) and correlation coefficient (R), Eqs. (1) and (2).

2 $${\text{RMSE}}=\frac{\overline{{({{\text{C}}}_{{\text{P}}}-{{\text{C}}}_{{\text{O}}})}^{2}}}{\overline{{{\text{C}}}_{{\text{O}}}} \overline{{{\text{C}}}_{{\text{P}}}}},$$

3 $$R=\frac{\overline{({C}_{O}-\overline{{C}_{O}})({C}_{P}-\overline{{C}_{P}})}}{{\sigma }_{{C}_{O}}{\sigma }_{{C}_{P}}},$$

where *C*_*o*_ mg/L is the actual data, ${C}_{P} \text{ mg/L}$ is the modeled data, $\overline{{C}_{O}}\text{ mg/L}$ is the average of the actual data, $\overline{{C}_{P}}\text{ mg/L}$ is the average of the modeled data, and *σ* is the standard deviation over the dataset. The criteria for statistically reasonable limits are 1 ≥ R > 0.8 and 0 ≤ RMSE < 1.5^15,21^.

### Sensitivity analysis

The sensitivity analysis is considered one of the most important methods necessary to understand the impact of sensitive parameters on the fate of cadmium within the treatment plant. In this study, the sensitivity parameter for the fate of cadmium in the Muharram Aisha sewage treatment plant, was found to be the mass of MLSS.

### Optimization processes

The previous studies that used to reduce cadmium concentrations in domestic and industrial wastewater have been typically very expensive, either in physical or chemical nature, and their use can typically have some harmful consequences. In this study, the most important sensitive factors to reduce cadmium concentrations were determined through the TOXCHEM model and the biological method was adopted. This method does not require any additional financial cost and instead depends on adjusting the appropriate concentration of cadmium in the aeration basin. In this study, the optimal concentration of MLSS was determined to bring cadmium concentrations within permissible limits in the treated water, taking into account the cadmium concentration in the sludge. After selecting the optimal concentration of MLSS, the plant was operated for a period of five months from 1/1/2023 to 1/5/2023, according to the parameters reported in Table 3, and the results were within the required limits, as shown in Table 7.

**Table 3:** Modifications of the default parameters for the Muharram Aisha WWTP in the TOXCHEM Model.

Unit process	Parameter	Unit	Default value	Calibration value	Validation value
	Flow rate	m^3^/day	50,000	5000	5000
	Suspended solids	mg/L	200	290	270
	VSS to SS ratio	%	75	78	77
	Oil/grease concentration	mg/L	0	39	41
	Temperature	°C	15	12	32
	SS removal efficiency	%	50	5	5
	Oil removal efficiency	%	80	91	93
	Sludge SS concentration	mg/L	10,000	0	0
	Flow rate of recovered oil stream	m^3^/day	500	0.1	0.1
	MLSS	mg/L	3000	1600	1400
	VSS to SS ratio	%	75	78	79
	Dissolved oxygen	mg/L	2	3	2.5
	Process air flow rate	m^3^/h	10,000	2500	2500
	Oxygen transfer efficiency	%	10	11	8
	SRT	day	10	25	25
	Effluent SS concentration	mg/L	10	6	10
	Sludge SS concentration	mg/L	6000	5500	5600
	Final solid concentration	%	40	90	92
	Filtrate/centrate SS concentration	mg/L	200	250	300

*AP*I American Petroleum Institute.

## Results and discussion

### The performance evaluation of Muharram Aisha WWTP

**Figure 2:**
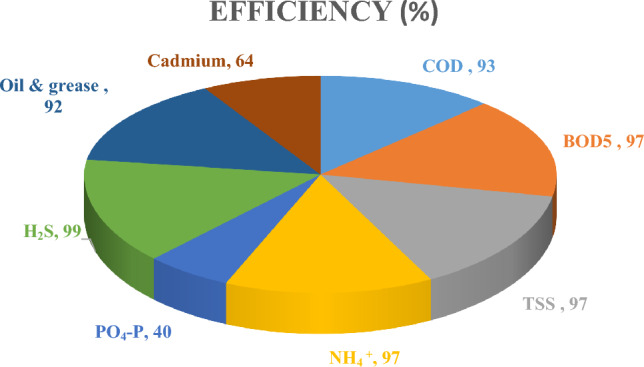
Muharram Aisha WWTP efficiency performance ratios.

The results for the Muharram Aisha treatment plant showed that it conformed to Iraqi specifications, as shown in Table 2 and Fig. 2, with the exception of cadmium, which was not among the required parameters. The percentage removals of COD, BOD_5_, TSS, NH_4_^+^, PO_4_-P, H2S, oil and grease, and cadmium pollutants were 93, 97, 97, 97, 40, 99, 92, and 64%, respectively. The results indicate that a significant proportion of the COD and BOD_5_ was removed, indicating good biodegradation, excellent oxidation in the presence of dissolved oxygen and good mixing inside the aeration basin, which contributed to a good stabilization process. These are important factors in reducing these pollutants, as indicated by Ref.^22^. The proportion of TSS removed was 97%, indicating an excellent sedimentation process and good flocculation of the biomass inside the sedimentation basins. Through the physical examination of the SVI, the indicators were good for the sedimentation process, and were within acceptable limits^23^. A good nitrification process was apparent through the oxidation processes of ammonia and its conversion to nitrate and the efficiency was found to be greater than 97%. Denitrification operations in this plant are not good due to the absence of an anoxic basin. An acceptable removal of phosphates of 40% was observed along with excellent removal of hydrogen sulfide gas at 99%, due to the oxidation and stripping processes that take place inside the aeration basin and the adsorption of this gas by the biomass. High oxidation increases the sulfate concentrations in the outgoing water due to the conversion of hydrogen sulfide gas into sulfate and the transformation of proteins and amino acids into stabilized compounds, including sulfates^15^. Good fat removal was observed, at 92%, due to the efficiency of the fat removal basin. Almost all the pollutants coming out of this plant are within the permissible limits, except for cadmium, which is a very dangerous indicator in the short and long term. Despite the 64% removal of this element, its concentrations remained high in the treated wastewater, which may lead to detrimental effects on human health if the outlet water is used directly for irrigation. Therefore, this problem has been targeted by researchers to find suitable solutions these can be successfully used to remove this harmful element.

### Model calibration and validation at the Muharram Aisha WWTP

**Figure 3:**
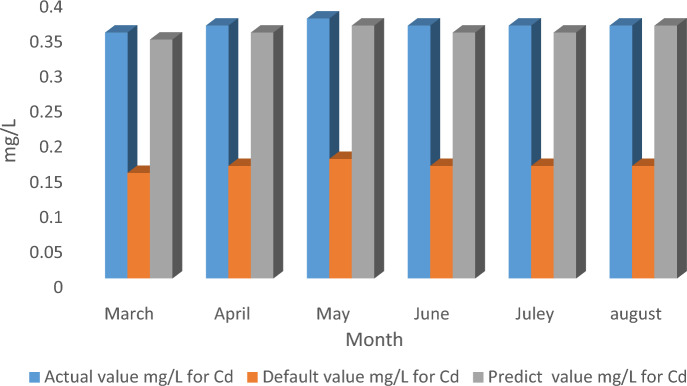
The calibration of the actual, predicted, and default TOXCHEM values (Muharram Aisha WWTP for 2022).

**Figure 4:**
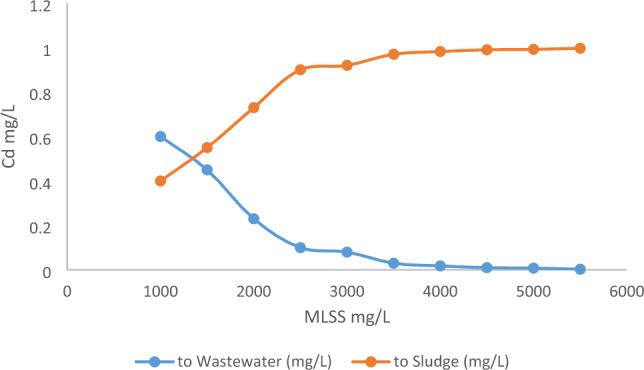
MLSS effect on Cd.

The model calibration for the Muharram Aisha WWTP can be achieved by adjusting the default values within the model that will have a direct effect on the results. After calibration, the results were always compared with the actual results of the plant outlet. The calibration in this study was set for an average of 6 months in the spring and summer seasons, using a TOXCHEM model^13^. Hence, the calibration was achieved by changing the default parameters in the model to the actual parameters obtained from the plant management during the abovementioned period, as shown Table 3. In this study, the model was initially run based on entering values of physical and operational conditions such as discharge, dimensions, and concentrations of pollutants and the parameters were adjusted until satisfactory results for R (0.81) and RMSE (0.0001) were obtained (Table 4). Table 3 and Fig. 3 show a comparison of the default, actual, and predicted values after suggested modifications. Default values within the model are usually adjusted due to the different conditions and nature of each plant and region. After the calibration was completed, the model was validated. Model validation can be defined as gaining an excellent fit between model expectations and a dataset that was not used in the model calibration within the required parameters^20^. The model was successfully validated by adopting six-month average output values for the fall and winter seasons and comparing these with the actual results as shown in Table 5. Figure 4 shows a comparison between the values for cadmium at the outlet of the plant for the actual, the default, and the predicted. The results show that slight changes in cadmium concentrations were observed across the seasons of the year.

**Table 4:** R and RMSE values before and after adjustment for calibration.

Month	Actual value mg/L for Cd	Default value mg/L for Cd	Predict value mg/L for Cd	R before calibration	R after calibration	Calibration limits for R	Ideal fit	RMSE before calibration	RMSE after calibration	Calibration limits for RMSE	Ideal fit
March	0.35	0.15	0.34	0.2	0.81	0.8–1	1	0.054	0.0001	≤ 1.5	0
April	0.36	0.16	0.35								
May	0.37	0.17	0.36								
June	0.36	0.16	0.35								
July	0.36	0.16	0.35								
August	0.36	0.16	0.36

**Table 5:** Fate summary for Cd.

Contaminant load	Mass (g/day)	% of total
Total incoming	13,000	100
To air	0	0
To wastewater	4832.44	37.1726
To sludge	8164.52	62.804
To oil	0	0
Removed in air treatment	0	0
Biodegraded	0	0

### Cd fate in Muharram Aisha WWTP

The mass load of cadmium at the Muharram Aisha WWTP is 13 kg/day. This load, along with the discharge inside the plant, is considered an indicator of the biological processes that take place inside the aeration basin. Previous studies have indicated that cadmium levels of 1 mg/L or lower are necessary to prevent the action of the bacteria from being inhibited, reducing their efficiency in terms of removing pollutants^24^. Table 6 shows the fate of cadmium at the Muharram Aisha WWTP. It was observed that 37% was lost in the treated wastewater and 63% remained adsorbed within the sludge. The results showed that the highest effect on the fate of cadmium in the treatment plant was the concentration of MLSS and its excess to the drying beds. The concentration of cadmium in the outgoing effluent wastewater was observed to be outside the permissible threshold limits for discharge as stipulated by Iraqi legislation. Therefore, a sensitivity analysis of the relevant system parameters was required to control cadmium concentrations in the treated wastewater. Cadmium was not affected by any biological decomposition processes or its spread in the air except for one process, which is its absorption by the biomass.

**Table 6:** Characteristics of Muharram Aisha WWTP after the optimization process.

Parameter	Inlet concentration	Outlet concentration	Standard Iraq (River Conservation Legislation No. 25 of 1967)
PH	7.2	7.1	7
COD (mg/L)	500	39	100
BOD_5_ (mg/L)	330	10	40
TSS (mg/L)	250	25	60
NO_3_ (mg/L)	0	49	50
NH_4_^+^ (mg/L)	22	0.1	1
PO_4_-P (mg/L)	5	1.8	3
H_2_S (mg/L)	35	ND	3
SO_4_ (mg/L)	400	450	600
Oil and grease (mg/L)	40	3	4
Cadmium (mg/L)	1	0.01	0.01

### Sensitivity analysis

Through the results of the mass balance and the fate of cadmium shown in Table 6, it was noted that the most important sensitive factor is the biomass concentration represented by the MLSS. The extended aeration system is affected by several factors, the most important of which is the F/M ratio. One possible definition of the F/M ratio is the amount of food (BOD_5_ concentration) to the ratio of microorganisms (MLVSS). This ratio in the extended aeration process is between 0.04 and 0.1. This ratio can be obtained from the equation below^25^:

4 $$ {\text{F}}/{\text{M Ratio }} = \, \left( {{\text{BOD}}_{{5}} {\text{in}}. \, \times {\text{ Qin}}.} \right)/\left( {{\text{MLVSS}} \times {\text{V}}} \right). $$

Using the above equation, it is possible to model the effects of high concentrations of MLSS to obtain the highest possible adsorption of cadmium without deviating from the limits of the f/m ratio. Figure 4 shows that an increase in the concentration of MLSS from 1000 to 5500 mg/L will lead to excellent reductions of cadmium in the treated wastewater with predicted concentrations approaching zero. The flocculation characteristic of the biomass, increases the adsorption of cadmium by the slime surrounding the cell membrane, attracting it and then absorbing it, which contributes to reduced cadmium concentrations in treated wastewater and a corresponding increase in sludge the concentrations. This is consistent with the study presented by Ref.^26^. When the MLSS concentration was increased to 4500 mg/L, the cadmium concentration was found to lie within acceptable limits.

### Optimization processes

In this study, the most important sensitive factor in the plant was identified, as the MLSS concentration, which was effective in reducing the cadmium concentration without any additional cost. The novelty of this study is the adoption of the biological method through increasing the concentrations of MLSS, leading to a concurrent reduction in cadmium concentrations without any effect on other factors. This was achieved via two approaches, the first theoretical and the second practical. After simulating the plant, the MLSS concentration was found to be 4500 and the f/m ratio was 0.09. After operating the plant for a period of five months and the adoption of Table 7 instead of 1, where the results of the plant are as shown in Table 6. It was observed that increasing the concentrations of MLSS, led to a slight increase in the output of the plant due to the increase in the mass load in the secondary sedimentation basins. In addition to the exit of cell debris as a result of endogenous respiration, which occurred as a result of increasing the concentration of MLSS in the aeration basin^12^, there is also an increase in the percentage of activated sludge from 50 to 100% which increased the concentration of MLSS in the aeration basin.

**Table 7:** Operational conditions of Muharram Aisha WWTP after optimization processes.

Parameter	Value
Flowrate (Q)	13,000 m^3^/day
Sludge retention tank (SRT)	28 days
Hydraulic retention time (HRT)	10 h
Mix liquor suspended solid (MLSS)	4500 mg/L
Food/microorganism (F/M) ratio	0.09 kg BOD/kg MLVSS day
Dissolved oxygen (DO)	2–3 mg/L
Return activated sludge (RAS)	100%
Sludge volume index (SVI)	110 mL/g

## Conclusion

Cadmium is one of the most toxic heavy metals affecting the environment and its presence within the effluent of waste water treatment plants is a cause for concern. The fate and removal of this element within the Muharram Aisha WWTP was simulated via a TOXCHEM model. In this study the following conclusions were obtained:

- Prior to analysis, the model was calibrated and validated for cadmium, with the adjustments leading to a mean square error (RMSE) and correlation coefficient (R) of 0.0001 and 0.81, respectively
- The mass balance of cadmium in the Muharram Aisha treatment plant was found to be 4832.44 g/day (37.1726%) in the treated wastewater and 8164.52 g/day (62.804%) in the sludge.
- The MLSS increased from 1500 to 4500 mg/L, and this reduced the concentration of cadmium in the wastewater from 0.36 to 0.01 mg/L
- The simulation results showed that cadmium is significantly affected by biomass and the TOXCHEM model effectively contributed to determining the optimal concentration of MLSS to be used in the plant for practical cadmium removal.
- It has been proven that heavy metals, especially cadmium, can be removed by increasing the MLSS concentration experimentally and theoretically.
- Following modelling and optimization, the f/m ratio was adjusted to 0.09 and effluent concentrations were within permissible limits for elemental cadmium, as well as for all other pollutants in the outgoing wastewater.

### Supplementary Information

Supplementary Tables.

## Data Availability

All data used in the current study will be available from the corresponding author on reasonable request.
