# Supplementary material for: Simulation study of a practical approach to enhance cadmium removal via biological treatment by controlling the concentration of MLSS
Source: Sci Rep. 2024 Jan 19;14:1714. doi: 10.1038/s41598-023-50843-5 (PMC10799035; doi:10.1038/s41598-023-50843-5)
Supplement: Supplementary file 1 — Supplementary Tables. [file 41598_2023_50843_MOESM1_ESM.docx]

**Supplementary Information**

**Simulation Study of a Practical Approach to Enhance Calcium Removal Via Biological Treatment by Controlling the Concentration of MLSS**

Basim K. Nile ^a^, Ahmed M. Faris ^b^, Hasan F. Alesary ^c^, Nadhir N. A. Jafar^d^, Hani K Ismail^e^, Muhammad Abdulredha^f^ , Maad F Al Juboury^f^, Waqed H Hassan^g,f^ , Luma M Ahmed^c^, Hussein Rasool Abid^h^, Stephen Barton^i^

**^a^**Engineering College, University of Kerbala, Karbala 56001, Iraq.

^b^Kerbala Sewerage Directorate, Kerbala 56001, Iraq.

**^c^** Department of Chemistry, College of Science, University of Kerbala, Karbala, 56001, Iraq.

^d^Pharmacy College/ Al-Zahraa University for Women, Karbala, 56001, Iraq.

**^e^** Department of Chemistry, Faculty of Science and Health, Koya University, Koya KOY45, Kurdistan Region-F.R., Iraq.

**^f^** Department of Civil Engineering, College of Engineering, University of Kerbala, Kerbala, 56001, Iraq.

^g^ University of Warith Al-Anbiyaa, Kerbala, 56001, Iraq.

^h^ Environment health Applied Medical Science college, University of Kerbala, Karbala, Iraq.

**^i^**Kingston University London, School of Life Sciences, Pharmacy and Chemistry, Kingston-Upon-Thames, Surrey, UK.

Table S1 below shows the calculation of the plant's efficiency for pollutants

| Parameter | Efficiency (%) |
| --- | --- |
| COD | 93 |
| BOD5 | 97 |
| TSS | 97 |
| NH4+ | 97 |
| PO4-P | 40 |
| H2S | 99 |
| Oil & grease | 92 |
| Cd | 98 |

Table S2 below shows the calibration process for the TOXCHEM model

| Month | Actual value | Default value | Predict value |
| --- | --- | --- | --- |
|  | mg/L | mg/L | mg/L |
|  | for Cd | for Cd | for Cd |
| March | 0.35 | 0.15 | 0.34 |
| April | 0.36 | 0.16 | 0.35 |
| May | 0.37 | 0.17 | 0.36 |
| June | 0.36 | 0.16 | 0.35 |
| Juley | 0.36 | 0.16 | 0.35 |
| august | 0.36 | 0.16 | 0.36 |

Table S3 below shows the effect of MLSS on cadmium in treated wastewater and its concentration in sludge.

| Aeration tank MLSS (mg/L) | to Wastewater (mg/L) | to Sludge (mg/L) |
| --- | --- | --- |
| 1000 | 0.6 | 0.4 |
| 1500 | 0.45 | 0.55 |
| 2000 | 0.23 | 0.73 |
| 2500 | 0.1 | 0.9 |
| 3000 | 0.08 | 0.92 |
| 3500 | 0.03 | 0.97 |
| 4000 | 0.018 | 0.982 |
| 4500 | 0.01 | 0.99 |
| 5000 | 0.008 | 0.992 |
| 5500 | 0.003 | 0.997 |

Table S4 below shows the samples collected from the plant management for a full year of incoming and outgoing wastewater and before the improvement process.

| Parameter (inlet concentration) | month1 | month2 | month3 | month4 | month5 | month6 | month7 | month8 | month9 | month10 | month11 | month12 |
| --- | --- | --- | --- | --- | --- | --- | --- | --- | --- | --- | --- | --- |
| COD (mg/L) | 480 | 495 | 520 | 510 | 500 | 505 | 450 | 430 | 560 | 510 | 550 | 500 |
| BOD5 (mg/L) | 305 | 350 | 355 | 310 | 285 | 285 | 255 | 245 | 345 | 335 | 350 | 360 |
| TSS (mg/L) | 240 | 245 | 260 | 265 | 255 | 235 | 250 | 230 | 280 | 255 | 245 | 250 |
| NH4+ (mg/L) | 22 | 23 | 21 | 20 | 22 | 21 | 19 | 19 | 24 | 25 | 23 | 23 |
| PO4-P (mg/L) | 5 | 6 | 5 | 6 | 4 | 4 | 5 | 5 | 5 | 4 | 6 | 5 |
| H2S (mg/L) | 30 | 30 | 25 | 30 | 35 | 40 | 45 | 40 | 45 | 35 | 30 | 35 |
| Oil & grease (mg/L) | 40 | 45 | 35 | 41 | 42 | 39 | 38 | 40 | 38 | 42 | 40 | 40 |
| Cadmium (mg/L) | 0.9 | 1 | 1.1 | 1 | 0.8 | 1.2 | 1.1 | 0.9 | 1 | 1.1 | 0.8 | 1 |
|  |  |  |  |  |  |  |  |  |  |  |  |  |
| Parameter (outlet concentration) | month1 | month2 | month3 | month4 | month5 | month6 | month7 | month8 | month9 | month10 | month11 | month12 |
| COD (mg/L) | 35 | 40 | 42 | 28 | 30 | 35 | 33 | 31 | 39 | 35 | 32 | 35 |
| BOD5 (mg/L) | 11 | 9 | 10 | 12 | 8 | 10 | 11 | 9 | 10 | 8 | 12 | 10 |
| TSS (mg/L) | 8 | 7 | 9 | 10 | 7 | 7 | 8 | 10 | 6 | 8 | 9 | 7 |
| NH4+ (mg/L) | 0.5 | 0.4 | 0.45 | 0.55 | 0.6 | 0.5 | 0.45 | 0.55 | 0.5 | 0.52 | 0.48 | 0.5 |
| PO4-P (mg/L) | 2 | 2.5 | 1.5 | 2 | 2.2 | 1.8 | 2 | 2.1 | 1.9 | 2 | 2 | 2 |
| H2S (mg/L) | ND | ND | ND | ND | ND | ND | ND | ND | ND | ND | ND | ND |
| Oil & grease (mg/L) | 3 | 3 | 4 | 2 | 3 | 2 | 4 | 3 | 3 | 4 | 2 | 3 |
| Cadmium (mg/L) | 0.37 | 0.35 | 0.35 | 0.36 | 0.36 | 0.36 | 0.36 | 0.36 | 0.38 | 0.34 | 0.35 | 0.37 |

Table S5 below shows the tests that were conducted by the research team for a period of five months after the process of improving the incoming and outgoing wastewater

| Parameter (inlet concentration) | month1 | month2 | month3 | month4 | month5 |
| --- | --- | --- | --- | --- | --- |
| COD (mg/L) | 490 | 510 | 500 | 510 | 490 |
| BOD5 (mg/L) | 330 | 350 | 280 | 310 | 350 |
| TSS (mg/L) | 250 | 255 | 245 | 260 | 240 |
| NH4+ (mg/L) | 22 | 23 | 21 | 20 | 22 |
| PO4-P (mg/L) | 4 | 6 | 5 | 5 | 4 |
| H2S (mg/L) | 25 | 35 | 25 | 30 | 35 |
| Oil & grease (mg/L) | 44 | 46 | 35 | 41 | 39 |
| Cadmium (mg/L) | 1 | 0.8 | 1.2 | 1 | 1.1 |
|  |  |  |  |  |  |
| Parameter (outlet concentration) | month1 | month2 | month3 | month4 | month5 |
| COD (mg/L) | 40 | 40 | 38 | 39 | 37 |
| BOD5 (mg/L) | 10 | 12 | 8 | 10 | 9 |
| TSS (mg/L) | 25 | 30 | 20 | 25 | 26 |
| NH4+ (mg/L) | 0.1 | 0.12 | 0.13 | 0.1 | 0.1 |
| PO4-P (mg/L) | 1.8 | 1.9 | 2 | 2 | 2.2 |
| H2S (mg/L) | ND | ND | ND | ND | ND |
| Oil & grease (mg/L) | 4 | 3 | 3 | 2 | 3 |
| Cadmium (mg/L) | 0.01 | 0.011 | 0.01 | 0.01 | 0.01 |
